# Optimized intrusion detection for IoT networks using Cauchy–Gaussian hybrid evolutionary feature selection

**DOI:** 10.1038/s41598-025-29884-5

**Published:** 2025-12-20

**Authors:** T. Saranya, S. Indra Priyadharshini

**Affiliations:** School of Computer Science and Engineering,Vellore Institute of Technology, Chennai,TamilNadu, 600127 India

**Keywords:** Active feature selection, Arithmetic optimization algorithm (AOA), Ensemble machine learning, Internet of Things (IoT), Intrusion detection system (IDS), KD-tree, Engineering, Mathematics and computing

## Abstract

The Internet of Things network is a prime target for attackers due to its vulnerabilities and the sensitive data it handles. Protecting these devices is critical, and an Intrusion Detection System serves as the first defence against breaches. While many intrusion classification methods exist, building low-complexity systems for IoT remains challenging. This paper introduces a novel method combining active feature selection and ensemble machine learning to address complexity issues for IDS in IoT. Specifically, a novel Cauchy–Gaussian genetic-arithmetic optimiser-driven variance-based active feature selection method is proposed. The proposed algorithm operates in two phases: in the first phase, the model learns active samples by representing them as a KD-tree based on feature variance; in the second phase, the Cauchy–Gaussian genetic-arithmetic optimiser uses the active samples to select relevant features. Cauchy and Gaussian distributions ensure diversity in population initialisation and prevent early convergence, enhancing population diversity in the initial phase. The proposed genetic arithmetic optimizer combines genetic and arithmetic operators; the optimizer balances exploration and exploitation, accelerating convergence while avoiding local minima. The proposed method is evaluated and validated on the CICIDS 2017 and IoTID20 datasets, demonstrating superior performance to conventional AOA and GA approaches. Moreover, active feature selection reduces the complexity of running wrapper methods by using active samples for feature selection. The proposed method achieved an accuracy of 99.88% and 99.72% with the Bagging algorithm, along with a low false positive rate of 0.000801 and 0.000165 with the CICIDS2017 and IoTID20 datasets.

**Supplementary Information:**

The online version contains supplementary material available at 10.1038/s41598-025-29884-5.

## Introduction

The Internet of Things (IoT) is a broad network of connected smart devices that communicate over the internet, found in homes, offices, healthcare, and more. This technology is rapidly transforming world and lifestyle by enabling real-time data sharing and smarter decision-making^1^. According to Fortune Business Insights’ 2023 IoT market report, the global IoT market was valued at USD 595.73 billion and is projected to rise to USD 714.48 billion in 2024, reaching USD 4,062.34 billion by 2032, with an anticipated compound annual growth rate (CAGR) of 24.3%^2^. However, the growing adoption of IoT devices and the increasing complexity of IoT network architectures have also introduced new security challenges and risks^3^. Most IoT devices lack essential built-in security mechanisms to defend against cyber-attacks; since they are typically designed for specific, limited functions, security considerations are often neglected, leaving them highly vulnerable to cyber threats^4^. An intrusion detection system (IDS) helps to increase network security by detecting hostile activity within a system. The incorporation of artificial intelligence (AI) techniques improves IDS capabilities, allowing for more accurate detection of new threats such as zero-day vulnerabilities. In IoT environments, IDS solutions need an efficient handling of a large volume of network logs with minimal computational overhead on resource-constrained devices. Consequently, researchers use techniques such as dimensionality reduction and normalisation to manage the increasing complexity of network logs in AI-based IDS systems^5^.

Feature selection removes noisy or redundant features while retaining the most relevant ones, reducing computational cost and improving model accuracy. It is generally categorised into filter, wrapper, and embedded methods^6^. The filter method evaluates statistical properties of data independently of any classifier, while the wrapper method assesses subsets using a specific algorithm for better accuracy. But the wrapper method is efficient and computationally intensive compared to filter-based methods. To perform efficient dimensionality reduction using the wrapper technique for datasets with many labelled samples, it’s crucial to balance computational efficiency and performance. Traditional feature selection methods reduce dimensionality using all available data, but as the dataset grows, random sampling is commonly used. However, random sampling is indiscriminate, ignores data characteristics. This work introduces active feature selection, which partitions data based on variance and samples representative instances to reduce noise and enhance feature accuracy and efficiency of feature selection^7^. To the best of current knowledge, this is the first application of active feature selection in an intrusion detection system (IDS).

In recent years, optimization algorithms have shown strong potential in addressing feature selection problems^8^. Various metaheuristics inspired by biological evolution, swarm intelligence, and arithmetic operations, newer algorithms such as the reptile search algorithm (RSA), red fox optimiser (RFO), and crayfish optimization algorithm (COA) may of the algorithm face issues like premature convergence and limited exploration in high-dimensional spaces. According to Wolpert and Macready’s No Free Lunch (NFL) Theorem^9^, no single optimiser performs best across all problems; hence, this research integrates two well-established optimisers to create a complementary hybrid. This study combines the genetic algorithm (GA) and arithmetic optimization algorithm (AOA). The choice of GA and AOA is based on their proven ability to balance exploration and exploitation together, providing a balanced and effective approach for IDS feature selection^10^.

To develop an effective IDS for IoT networks, this study used the CICIDS2017^11^ and IoTID20^12^ datasets with essential preprocessing. Large training data were sampled using HVS sampling, followed by Cauchy–Gaussian genetic-arithmetic optimizer (CG-GAO) feature selection for dimensionality reduction. Ensemble classifiers were then employed for robust attack detection. On CICIDS2017, the state-of-the-art Hybrid OSMOGA (2024)^13^ achieved 99.43% accuracy, while the proposed CG-GAO achieved 99.88%, improves by 0.45%. On IoTID20, the IDSBPSO + RF (2022)^14^ achieved 99.84%, and the proposed method achieved 99.72%, offering comparable accuracy with fewer features.

The key contributions of this article are:

1. Active Feature Selection and KD Tree Construction**:** A KD Tree-based active feature selection approach is employed to select relevant samples from large training datasets. The KD Tree is constructed based on feature variance to reduce redundancy.
2. Population initialisation using Cauchy and Gaussian distributions enhances diversity by combining broad and balanced variations. This ensures wide exploration, prevents premature convergence, and helps the algorithm avoid local optima, and for finding the global optimum^15^.
3. Hybrid GAO Algorithm and Feature Set Reduction: The proposed optimization algorithm combines the mutation, crossover, and selection operators of the genetic algorithm (GA) with the arithmetic optimization algorithm’s (AOA) exploitation mechanisms to achieve enhanced exploration–exploitation balance. This hybrid GAO efficiently reduces high-dimensional feature spaces and improves classifier performance.
4. Ensemble Algorithms for IDS Implementation: To further increase the effectiveness of the intrusion detection system (IDS) ensemble algorithms is added with the CG-GAO Active feature selection is added for robust and generalised classification.
5. Comprehensive Comparative Evaluation**:** The proposed method is compared with baseline AOA and GA algorithms, supported by an ablation study, statistical significance testing, and cross-fold validation to ensure generalisation and reliability of IDS performance.

The paper is structured as follows: section “Literature survey” reviews the literature on different types of dimensionality reduction techniques used in IoT IDS over the years. Section “Motivation” is about the motivation for the proposed IDS with CG-GAO. Section “Proposed active feature selection” details the proposed methodology section “Results and discussion” provides the experimental results and detailed discussions, while section “Conclusion” wraps up the paper and outlines possible directions for future research.

## Literature survey

IoT devices are central to smart applications, which generate large amounts of sensitive data^16^, but their limited processing capability and lack of standard security protocols make them vulnerable. Signature-based IDS detects only known attacks. Anomaly-based IDS distinguishes normal and abnormal traffic^17^, thereby detecting unseen attacks better.IDS often struggle with IoT scalability and computational limits, leading to research on Efficient and lightweight IDS frameworks. Recent studies highlight diverse methods for efficient feature selection to lower the computational overhead and to improve the efficiency of IDS in IoT environments.

### Filter-based methods

An Intrusion Detection System presented in^17^, the CL-GAN framework integrates fast correlation-based filter (FCBF) and Information Gain for feature selection with convolutional neural networks (CNN), long short-term memory (LSTM), and generative adversarial networks (GAN) to detect DoS and botnet attacks. This model was assessed using the NSL-KDD, CICIDS2018, and Bot-IoT datasets and achieved up to a 5% increase in accuracy. In^18^ stacked ensemble IDS for IoT devices with the Fisher Score algorithm for Feature selection. Tested on NB-IoT and UNSW-NB15 datasets, achieved 99.68% accuracy.

### Wrapper-based methods

In^19^, bijective soft set is used to find optimal features using the CorrACC metric. Evaluated on the BoT-IoT dataset, it achieved over 95% accuracy with the C4.5 decision tree and Random Forest. In^20^, decision tree with recursive feature elimination (RFE) achieved 99.21% accuracy on NSL-KDD and 99.94% on CICIDS 2017, identifying key features and reducing computational costs.

### Hybrid methods

In^21^, K-nearest neighbors (K-NN) classifier with hybrid feature selection using PCA, statistical tests, and genetic algorithms,tested on Bot-IoT dataset, it achieved 99.99% accuracy and prediction time was reduced from 51,182 s to under a minute. In^22^ a hybrid feature selection m ethod combines the residue number system (RNS),Bat algorithm and PCA. K-nearest neighbors and Naïve Bayes are employed for classification, tested on NSL-KDD dataset, the Bat with RNS obtained 99.15% accuracy,98.09% detection rate, and a training time of 55.65 s.

### Metaheuristic algorithm-based methods

Metaheuristic algorithms have emerged as powerful tools in the design of intrusion detection systems (IDSs), particularly for addressing two critical challenges: feature selection (FS) and hyperparameter optimization. These algorithms explore high-dimensional search spaces by imitating biological or natural processes, which makes them perfect for cybersecurity applications where detection accuracy and model generalization are impacted by the selection of optimal features. The recently introduced Hazelnut Tree Search (HTS) algorithm (which incorporates growth, fruit scattering, and root spreading operators to balance exploration and exploitation)^23^. The Chaotic Election Algorithm (CEA)^24^ (an improved version of the Election Algorithm that incorporates chaotic positive advertisement and migration operators to enhance global search and maintain population diversity),the Chaotic local search–levy flight distribution (CLS–LFD)^25^ algorithm (which optimizes resource allocation by integrating chaotic search behavior with Levy flight for improved convergence—to optimize deep learning and ensemble classifiers. The Locally Weighted Salp Swarm Algorithm (LWSSA)^26^ enhances SSA with local weighting and mutation mechanisms, improving convergence and diversity; when combined with XGBoost, it achieved superior predictive accuracy in CVD risk assessment. Table 1 discusses some recent studies that use metaheuristic optimization algorithms for IDS from 2023 to 2025.

**Table 1:** Overview of recent metaheuristic-based intrusion detection systems (2022–2025).

Year	Metaheuristic algorithm used	Key contribution
2022 ^27^	Time-based Leadership Particle Swarm-based Salp Algorithm (TPSOSA)	Proposed TPSOSA achieved higher accuracy and better feature reduction than other metaheuristics, confirmed by Friedman and Wilcoxon tests Tested on CEC 2017, CEC2008lsgo, NSL-KDD and 19 datasets
2023 ^28^	pigeon-inspired optimization (PIO) + Local Search (tabu + hill climbing)	LS-PIO enhances PIO with Tabu and Hill Climbing for feature selection. Achieved high accuracy and F1-score across BoT-IoT, UNSW-NB15, NSL-KDD, and KDDCUP99 datasets
2024 ^29^	squirrel search optimization (SSO)	Combines SSO with Bengio Nesterov Momentum CNN (BNMCNN) to enhance precision (0.96) and reduce computational time on NSL-KDD and CICIDS 2017 datasets
2023 ^30^	Gaussian Mutation and Shrink Moth-Flame Optimization (GMSMFO)	An improved version of MFO using Gaussian mutation and shrink strategy to enhance diversity and balance exploration–exploitation. Tested on CEC 2017 benchmarks and NSL-KDD. GMSMFO achieved higher accuracy and fewer features than other metaheuristics, confirmed by Friedman and Wilcoxon tests
2023 ^31^	capuchin search algorithm (CapSA)	CNN-CapSA integrates CNN feature extraction with CapSA-based selection, improving accuracy and lowering computational cost across NSL-KDD, BoT-IoT, KDD99, and CICIDS 2017 datasets
2025 ^32^	genetic algorithm (GA)	GA-tuned ensemble of deep transfer learning models (NFIoT-GATE-DTL IDS) for NetFlow-based intrusion detection, achieving 100% accuracy across 15 attack types and outperforming other optimizers
2025 ^33^	grey wolf optimization (GWO) + Quantum Binary bat algorithm (QBBA) (Hybrid: GWQBBA)	Hybrid GWQBBA model for feature selection, achieving 98.5% accuracy with Random Forest on UNSW-NB15 using only 12 features, while maintaining high sensitivity and F-measure across classifiers
2024 ^34^	golden jackal optimization algorithm (GJOA) with salp swarm algorithm (SSA)	GJOADL-IDSNS, a hybrid IDS combining GJOA-based feature selection with SSA-tuned attention-based BiLSTM, achieves superior accuracy and robustness on benchmark datasets
2024 ^35^	coati optimization algorithm (COA)	ID-COADL, an IDS for VANETs, combines COA-based hyperparameter tuning with a Deep Belief Network. Achieved improved detection and classification
2024 ^36^	artificial bee colony (ABC) with fuzzy Temporal Rules	FT-ABC-CNN, a hybrid IDS integrates fuzzy temporal rules, ABC optimization,for feature selection and CNN to enhance detection accuracy and minimise false positives.
2024 ^37^	chaotic honey badger optimization (CHBO)	Introduced a CHBO-based feature selection with Dugat-LSTM classifier for IDS, achieving 98.76% and 99.65% accuracy on TON-IoT and NSL-KDD datasets
2025 ^38^	Enhanced whale optimization algorithm (EWOA)	Utilized LDA for dimensionality reduction and EWOA for feature selection, combined with an RF-XGBoost, achieving 96.08% accuracy on the NSL-KDD dataset

## Motivation

Intrusion Detection Systems (IDS) for the Internet of Things (IoT) face significant hardships due to the large and noise present in log data, both in terms of features and instances. While wrapper-based feature selection methods can provide better performance by tailoring features to specific algorithms, their high computational cost makes them impractical for large IoT datasets. Filter models are more efficient but may miss important feature interactions. A promising approach to overcome the above difficulties is active feature selection, which selects representative instances, reducing the need to evaluate the entire dataset. However, from the extensive literature survey research on applying active feature selection to optimize wrapper models for IDS in IoT systems remains unexplored. Moreover, while biological evolutionary algorithms often excel in optimization problems, they are not universally effective. Researchers have noted that these methods can struggle with increased complexity and dimensionality, in line with the no-free-lunch (NFL) theorem, which claims that no one optimization algorithm excels across all types of problems^10^. Therefore, this research proposes a novel hybrid optimization approach based on active feature selection, integrating mutation, crossover, selection operators of genetic algorithm (GA) exploration, and exploitation operators of arithmetic optimization algorithm (AOA) with population initialization using Cauchy and Gaussian distribution. The initialization of the population in an optimization algorithm significantly influences the entire optimization process. To enhance the optimization process, the Cauchy–Gaussian distribution is used for population initialization. The Cauchy distribution aids in exploring outliers, while the Gaussian distribution helps to avoid extreme or irrelevant regions. This hybrid method addresses the complexity of wrapper methods, overcomes AOA’s limitations such as local search weaknesses, the proposed method provides a balance between exploration and exploitation phases and it overcomes the genetic algorithm weakness of slow convergence. Moreover, the proposed method will be an effective strategy for finding optimal solutions in complex, high-dimensional feature selection problems.

## Proposed active feature selection

**Fig. 1:**
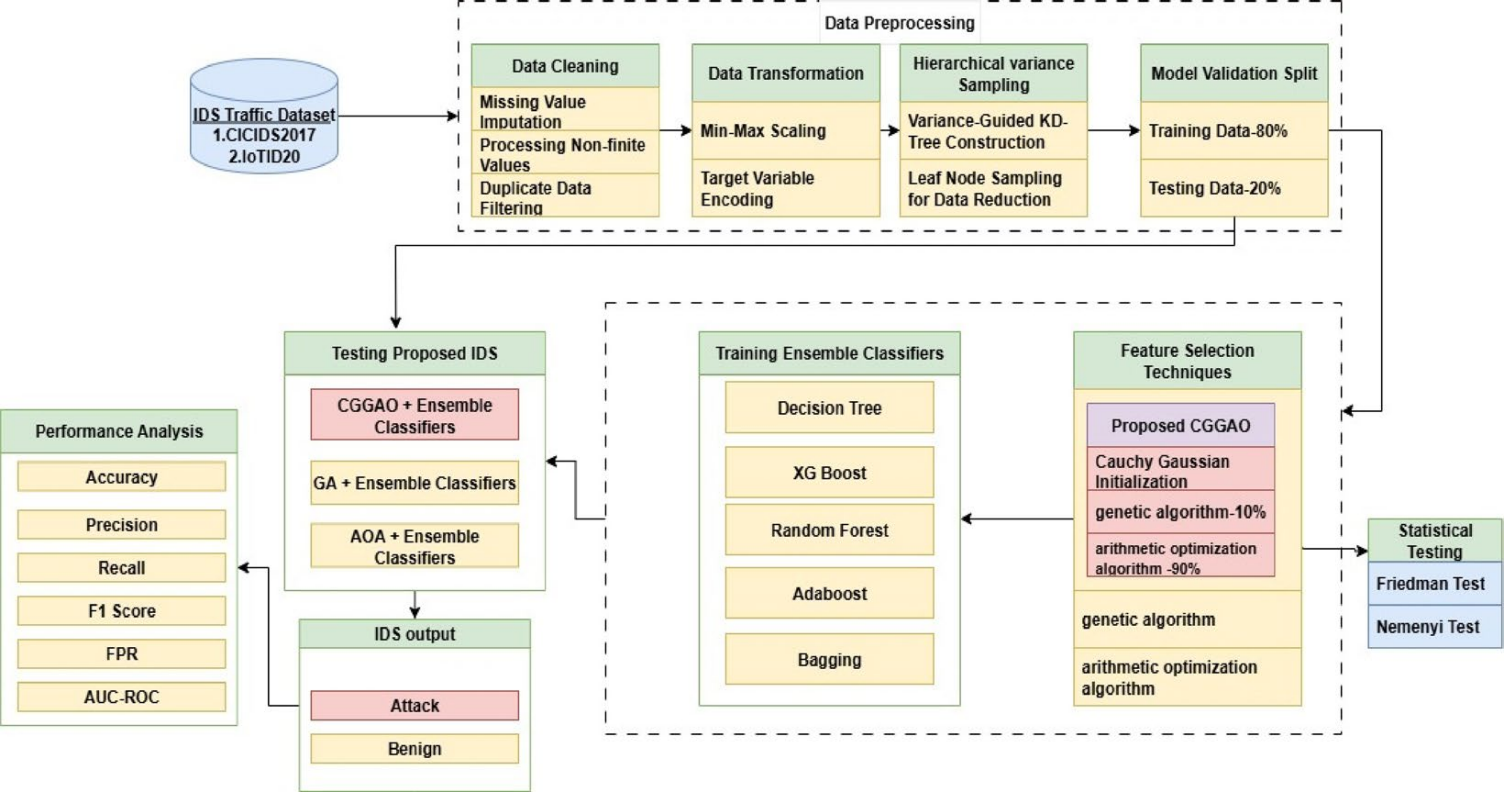
Architecture diagram of proposed intrusion detection system.

This section outlines the steps involved in intrusion detection system (IDS) utilising the novel CG-GAO-based active feature selection with an ensemble algorithm. Figure 1 shows the IDS architecture.

### Data pre-processing

Machine learning models need clean, well-structured data that is free from noise and redundancy for effective model training. Inconsistencies in raw log data, such as null values, duplicates, and irregular formats, affect model performance and result in poor classifications. A strong pipeline for data preprocessing is necessary to shape the data before training the classifier.

#### Handling null and duplicate values

To guarantee data quality, prevent bias, and preserve dependable model performance, handling null and duplicate values is crucial. To make the traffic data complete and balanced, null values (NaN) are filled with the class-wise average. Infinite values, resulting from operations like division by zero, are first converted to NaN and then replaced with the class-wise maximum to correct extremes. Negative values are converted to NaN and filled with the minimum positive value of each class, to make all features non-negative, and to make the dataset consistent for training the Classifier.

#### Feature normalisation

Normalisation ensures that all features are scaled uniformly, thereby improving model performance and convergence. Min–Max normalisation is widely used as it preserves data distribution, handles sparse datasets, and suits algorithms sensitive to feature scales. It is also less affected by outliers. This method scales values between 0 and 1 using the formula in Eq. (1)^39^.

1 $${\text{n}}_{{\text{i}}} ^{\prime } = \frac{{{\text{n}}_{{\text{i}}} {\text{ - N}}_{{{\text{min}}}} }}{{{\text{N}}_{{{\text{max}}}} {\text{ - N}}_{{{\text{min}}}} }}$$

Here, ${\text{N}}_{\text{min}}$ and ${\text{N}}_{\text{max}}$ are the lower and the upper bound values of the attribute N. The original and feature’s normalized value n, are indicated by ${\text{n}}_{\text{i}}$ and ${\text{n}}_{{\text{i}}} ^{\prime }$ respectively.

#### Target variable encoding

As the proposed methodology is for binary classification, the target variable of the attack datasets is encoded to Attack as 1 and Normal as 0 using Label encoding, and any other features that have categorical columns are also encoded using Label encoding.

### Phase-I

In phase I, a hierarchical variance-based KD-tree is used to sample the preprocessed data by identifying the most representative instances. Unlike conventional random sampling, this method utilises selective sampling, where the KD-tree partitions the dataset based on dissimilarity metrics^40^. The KD-tree structures the data in a multidimensional space, recursively selecting features with the highest variance for splitting. This process continues until the defined bucket size is reached, effectively capturing the original data distribution. A subset of data with similar statistical properties is grouped in each leaf of the KD-tree. This technique effectively reduces the dataset size by choosing balanced, variance-representative samples from each leaf. To maintain class balance, stratified sampling is used within each leaf node, selecting equal numbers of attack and benign samples whenever both classes are present. This approach preserves the data distribution while maintaining local class representativeness, which improves the computational efficiency and dependability of the feature selection algorithm^40^.

**Algorithm 1:**
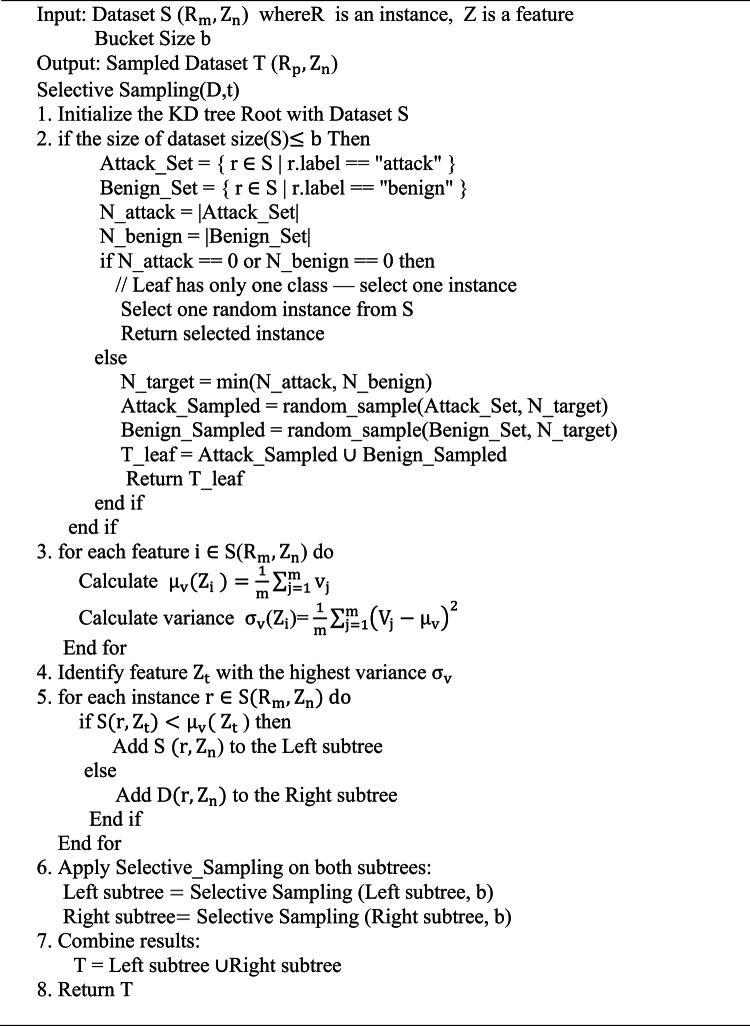
Hierarchical Variance Sampling using KD-Tree(HVS)

### Phase-II

In the second phase, feature selection using a novel Cauchy–Gaussian Genetic-Arithmetic Optimiser (CG-GAO) is applied.CG-GAO improves the solution of population initialization by using Cauchy and Gaussian distributions, where the Cauchy distribution helps in exploring outliers, and the Gaussian distribution provides a refined search around potential regions. In the proposed hybrid algorithm, the genetic algorithm is chosen for its global search ability, population diversity and generalizability across different optimization problems. However, GA has slow convergence and gets stuck in local optima. To overcome this, the arithmetic optimization algorithm (AOA) is combined in later iterations after good exploration for better local search and faster convergence, creating a good balance between exploration and exploitation.

The process starts by initialising a population P of N individuals, where two different groups are obtained using Cauchy and Gaussian distributions. These groups are then combined together to form a hybrid population.

2 $${P}_{{Cauchy}_{j}}={B}_{j}+Cauchy\left({\gamma }_{c},{x}_{0}\right)\times ({T}_{j}-{B}_{j})$$

3 $${P}_{{Gaussian}_{i}}={B}_{i}+N({\mu }_{n},{{\sigma }_{n}}^{2})\times ({T}_{j}-{B}_{j})$$

4 $$P_{j} = {\text{Concatenate}}\;(P_{Cauchy} ,P_{Gaussian} )$$

where ${T}_{i}$ = 1 and ${B}_{i}$ = 0 represents the upper and lower bounds of the search space, respectively. And $Cauchy\left({\gamma }_{c},{x}_{0}\right)$ represents sampling a random value from a Cauchy distribution, with scale parameter ${\gamma }_{c}$ and location parameter ${x}_{0}$. And $N({\mu }_{n},{{\sigma }_{n}}^{2})$ is a random value from the Gaussian (normal) distribution with mean ${\mu }_{n}$ and variance ${{\sigma }_{n}}^{2}$.

Each agent is converted into a binary representation to find the selected features. The Binary transformation rule:

5 $$BP_{{ij}} = \left\{ {\begin{array}{*{20}l} {1,} \hfill & {if\;P_{{ij}} > 0.5} \hfill \\ {0,} \hfill & {otherwise} \hfill \\ \end{array} } \right.$$

After removing features with zeros in the binary vector B, the fitness score is then calculated using:

6 $${Fit}_{i}=\beta \times {C}_{i}+\left(1-\beta \right)\times \left(\frac{\left|{BP}_{i}\right|}{{n}_{f}}\right)$$

In the Eq. 6, $\beta \in \left[\text{0,1}\right]$ is a weighting factor between the two components. Here, ${n}_{f}$ is the total number of features, $\left|{BP}_{i}\right|$ is the number of features selected, and ${C}_{i}$ is the classification error found using the K-Nearest Neighbors (KNN) algorithm.

The optimal solution ${P}_{b}$ is identified, after that the remaining agents are iteratively tuned using either genetic algorithm (GA) operators or Arithmetic Optimization Algorithm (AOA) mechanisms. This iterative process continues till the predefined maximum number of iterations ${I}_{t}$ is reached, in accordance with the update strategy:

7 $$P_{i} = \left\{ {\begin{array}{*{20}l} {GA\;phase} \hfill & {if\;t < 0.2 \times t_{max} } \hfill \\ {AoA \;Phase} \hfill & {Otherwise} \hfill \\ \end{array} } \right.$$

After reaching the stopping condition t = ${I}_{t}$ the individual with the best fitness score is chosen. The test set is then refined based on the selected features from this population, and classification performance is evaluated using the updated test set.

#### Genetic algorithm (GA phase)

Genetic algorithms (GA) are optimization methods that iteratively improve a population of candidate solutions. The process involves three main stages: selection, crossover, and mutation, and continues until a predefined termination criterion is satisfied.

*Selection*: Individuals are selected according to their fitness levels, where the roulette wheel selection method assigns a probability ${V}_{rs}\left({P}_{i}\right)$ to each candidate ${u}_{i}$ based on fitness score $f({P}_{i})$:

8 $${V}_{rs}\left({P}_{i}\right)=\frac{f({P}_{i})}{\sum_{j=1}^{m}{P}_{j}}$$

*Crossover*: Two parent solutions are combined to produce offspring. The Blend crossover technique generates new individuals using ${P}_{b}$ with the blending coefficient ${\alpha }_{g} set to 0.5$:

9 $$P_{i}^{1} = {\text{min}}\left( {P_{i}^{1} ,P_{i}^{2} } \right) - \alpha_{g} P_{b}$$

10 $$P_{i}^{2} = {\text{max}}\left( {P_{i}^{1} ,P_{i}^{2} } \right) - \alpha_{g} P_{b}$$

11 $$P_{b} = \left| {P_{i}^{1} - P_{i}^{2} } \right|$$

*Mutation*: Gaussian Mutation^41^ introduces small random perturbations to an individual by utilizing a Gaussian distribution with a standard deviation of σ = 0.1.

12 $${\text{mutate }}(P_{{ib}} ) = \times \left( {{\text{1}} + {\text{gaussian}}\left( \sigma \right)} \right)$$

These steps are iteratively repeated to improve the population until the algorithm converges.

#### Arithmetic optimization algorithm (AoA phase): initialization phase

Before the AOA process begins, it is necessary to compute the math optimizer acceleration operator (MOA) and the math optimizer probability (MOP). The MOA function governs the decision of whether the search will focus on exploration or exploitation, based on the value of the math optimizer accelerated (MOA) function^42^. The MOA function is defined as follows:

13 $${\text{MOA}}(I_{c} )\, = \,MOA_{min} + I_{c} \times \left( {\frac{{MOA_{max} - MOA_{min} }}{{I_{m} }}} \right)$$

In this context, ${I}_{c}$ refers to the current iteration, while ${I}_{m}$ indicates the maximum number of iterations. ${MOA}_{max}$ and ${MOA}_{min}$ represent the upper and lower bounds of the Acceleration function, respectively.

The math optimizer probability (MOP) is used to control the degree of exploitation during the optimization process. It is calculated using Eq. 14:

14 $${\text{MOP}}(I_{c} ) = {1} - \frac{{I_{c}^{{\frac{1}{\alpha }}} }}{{I_{m}^{{\frac{1}{\alpha }}} }}$$

where the sensitive parameter α is set to five and α influences the accuracy of exploitation across the iterations.

Exploration Phase

The Division (D) and Multiplication (M) operators are used during the exploration phase to explore the search space, help in improving diversity and prevent premature convergence.

15 $$P_{{i,j}} \left( {I_{c} + 1} \right) = \left\{ {\begin{array}{*{20}l} {best(P_{j} ) \div (MOP + \epsilon ) \times ((T_{i} - B_{i} ) \times \mu + B_{i} ,} & {r2 < 0.5} \\ {best(P_{j} ) \times MOP \times ((T_{i} - B_{i} ) \times \mu + B_{i} ,} & {otherwise} \\ \end{array} } \right.$$

where $best({P}_{j})$ is the best solution found, $\epsilon$ is the smallest integer number to prevent division by zero, ${T}_{i}$ and ${B}_{i}$ are maximum and minimum bounds of the current position help in regulating the search process. The parameter µ, set to 0.5, is used to fine-tune the search process.

Exploitation Phase

In the exploitation phase, the optimizer focuses on local refinement by applying the Subtraction (S) and Addition (A) operators. These operators decrease the solution diversity, enabling the efficient convergence towards the optimal solution.

16 $${Q}_{i,j}\left({I}_{c}+1\right)=\left\{\begin{array}{l}best\left({P}_{j}\right)-MOP\times (({T}_{i}-{B}_{i})\times \mu +{B}_{i}, r3 < 0.5 \\ best\left({P}_{j}\right)+MOP\times (({T}_{i}-{B}_{i})\times \mu +{B}_{i}, otherwise\end{array}\right.$$

**Algorithm 2:**
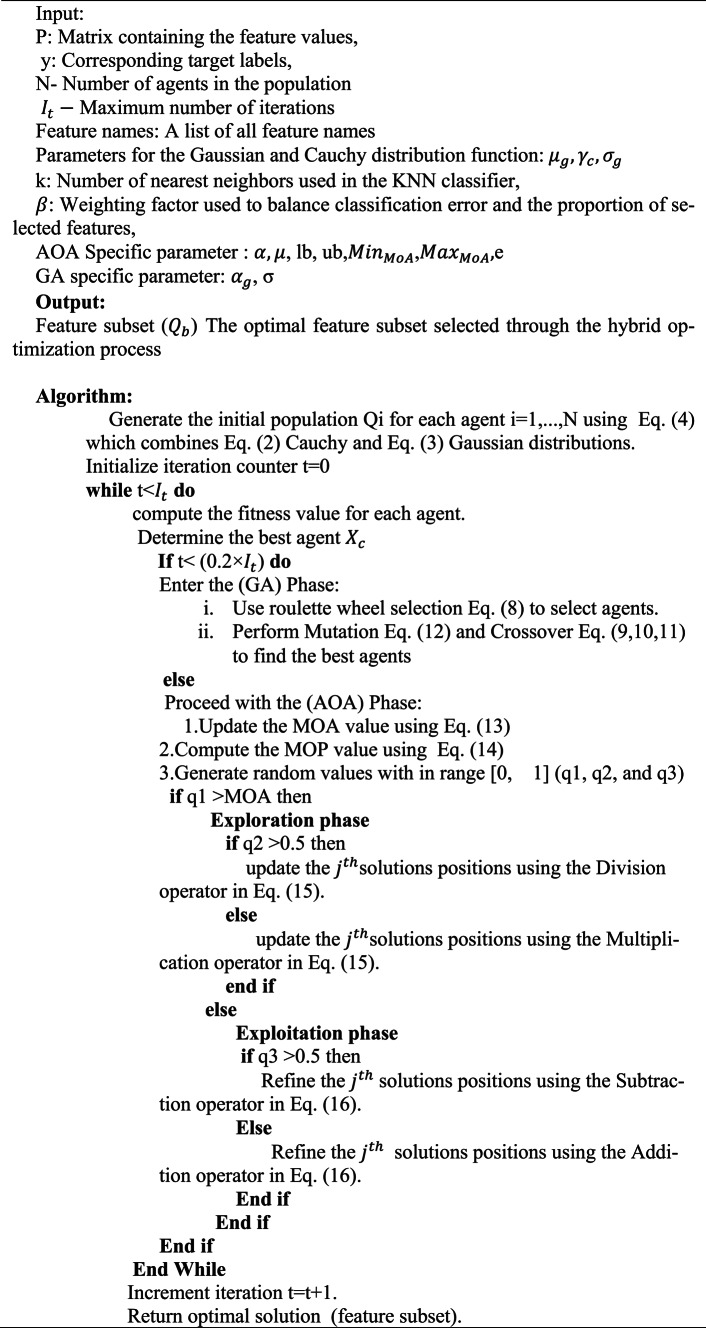
CG-GAO Feature Selection Algorithm

### Classification

Machine learning (ML) comprises intelligent models that learn from data to detect patterns and make decisions without explicit programming. Ensemble ML techniques improve IDS performance by combining multiple learners. In the proposed IDS, attack classification is carried out by active feature selection and ensemble classifiers such as Random Forest, AdaBoost, XGBoost, Decision Tree and Bagging Classifier.Each model has its own strengths like Random Forest reduces overfitting by combining multiple trees; AdaBoost and XGBoost improve weak learners through iterative learning^32^; and Bagging increases accuracy by lowering variance, making it suitable for changing network conditions^43^. The performance of these classifiers is assessed using standard evaluation metrics to support the claim of efficient intrusion detection.

## Results and discussion

This section presents the results of the proposed Intrusion Detection System (IDS), the experimental setup and the evaluation of the active feature selection method, including HVS sampling results and CGGAO feature selection results. Then, performance analysis of ensemble classifiers, an ablation study, comparison of the proposed with state of art techniques and crossfold validation results are discussed to highlight the performance of the proposed Intrusion Detection framework.

### Simulation environment

The proposed intrusion detection system (IDS) performance was evaluated using two benchmark datasets: CICIDS2017^11^ and IoTID-20^12^. The proposed CGGAOIDS is experimented with using the Google Colab environment. Experiments were conducted on a system with an Intel Core i5 processor (6 cores, 3.4 GHz) and 16 GB RAM.Key libraries such as NumPy, Pandas, Keras, TensorFlow, and Scikit-learn ensure better data handling, feature selection, ensemble model training, and performance evaluation.

#### Dataset description

The CICIDS2017 dataset is a benchmark dataset for network intrusion detection, simulates real-world traffic has about 2.8 million records and 83 features. It includes labelled records of six major attack categories and 14 subtypes. The dataset is known for its quality and detailed labels. About 19.70% of the records are attack traffic, and the remaining 80.30% are normal traffic^44^

The second dataset, IoTID20, records network activity in a smart home environment with connected devices such as AI speakers, Wi-Fi cameras, smartphones, and laptops. It includes different types of IoT attacks like DDoS, Mirai, DoS, ARP Spoofing, and Man-in-the-Middle. In this setup, devices like AI speakers and cameras served as targets, while other devices generated attack traffic. The dataset has 86 features and was created using CICFlowMeter, with labels assigned based on IP behaviour. It contains 40,073 normal records and 585,710 attack records^45^.

### Cleaning and preparation for IDS evaluation

To evaluate the proposed framework, the CICIDS2017 and IoTID20 dataset was cleaned and prepared. In CICIDS2017 columns, such as Flow ID, Source IP, Destination IP, Timestamp, Source Port, and Destination Port and in IoTID20 features such as pkSeqID, Category, Subcategory, Timestamp, Src IP, Dst IP, Src Port, and Dst Port are removed to avoid redundancy and data leakage. Missing or infinite values in both datasets (NaN or Infinity) were replaced with zeros, and columns with a large number of missing entries were removed. Categorical features are encoded into numerical values, and the target variable is converted into a binary format, labelling benign traffic as 0 and attacks as 1. Min–Max normalization is applied to scale the numerical features between 0 and 1. After preprocessing, the CICIDS2017 dataset has 78 columns and around 2.8 million records with 2,272,771 benign and 532,976 attack samples and the IoTID20 dataset contains 78 features and about 625,783 records with 40,073 benign and 585,710 attack samples.

### Hierarchical variance sampling (HVS) via KD-tree construction

After data preprocessing, both the CICIDS2017 and IoTID20 datasets were sampled using the Hierarchical Variance Sampling (HVS) algorithm, as detailed in Algorithm 1. The sampled distributions before and after HVS are listed in Table 2, while the configuration parameters used in HVS, including bucket size, target sample size, samples per leaf, and number of leaf nodes, are tabulated in Table 3. Figures S1 to S4 of Supplemenary shows the PCA Projection of data before and after sampling.

**Table 2:** Dataset summary before and after hierarchical variance sampling (HVS).

Dataset	Original samples	Benign	Attack	After HVS	Benign sampled	Attack sampled
CICIDS2017	2,805,747	2,272,771	532,976	56,824	28,412	28,412
IoTID20	625,783	40,073	585,710	20,235	10,117	10,118

**Table 3:** Parameters and configuration settings for hierarchical variance sampling via KD-tree.

Parameter	Value	Description
bucket_size	100	The number of records per leaf node. Controls the minimum size of the leaf node before sampling occurs
sampled_rows	List (populated during sampling)	A collection used to store sampled rows from each leaf node as the recursive process progresses
Depth	20	The maximum recursion depth for splitting the dataset, limiting splits to prevent overfitting and excessive granularity
Variances	Calculated during recursion	The computed variance of each feature (column) in the dataset, used to determine the optimal feature for splitting
split_column	Determined during recursion	The feature (column) with the highest variance, selected for dividing the dataset into two subgroups
split_value	Mean of the Split column	The average value of the selected column used as the threshold to partition the dataset into left and right subtrees
left_subtree	Data Frame (subset)	The subset of records where the values in the selected column are less than the split value
right_subtree	Data Frame (subset)	The subset of records where the values in the selected column are greater than or equal to the split value

### Feature selection via Cauchy–Gaussian genetic algorithm optimization (CG-GAO)

**Fig. 2:**
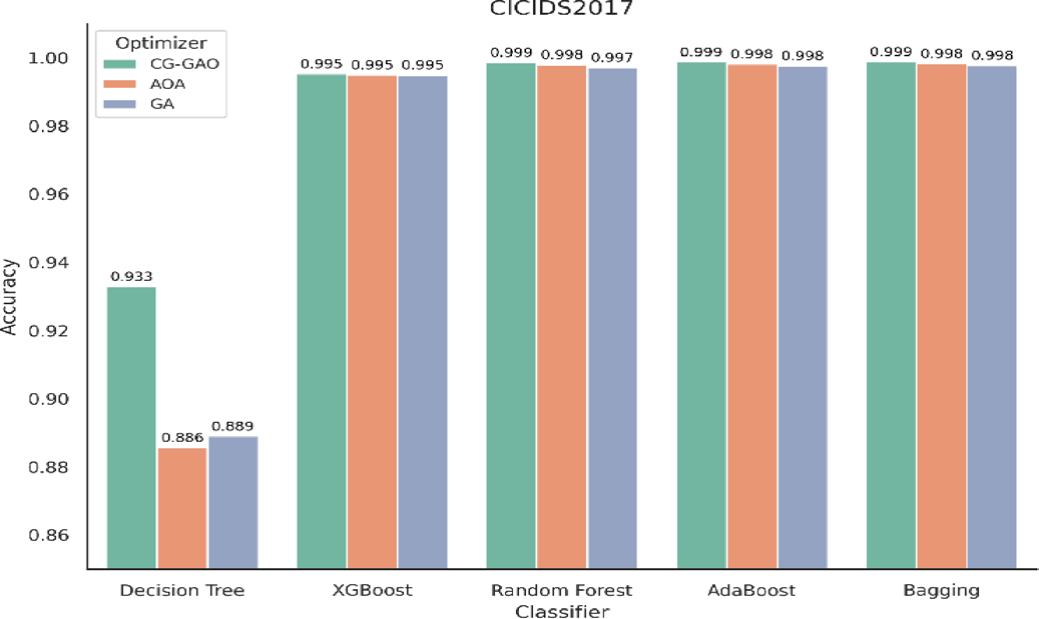
Accuracy of ensemble classifiers with feature selection techniques on CICIDS2017 dataset.

**Fig. 3:**
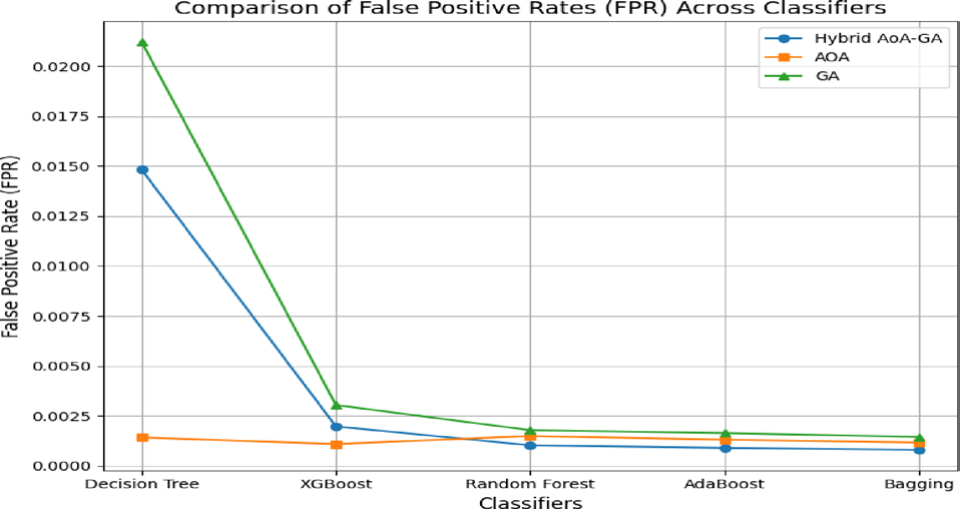
False positive rate of ensemble classifiers with feature selection techniques on CICIDS2017 dataset.

**Fig. 4:**
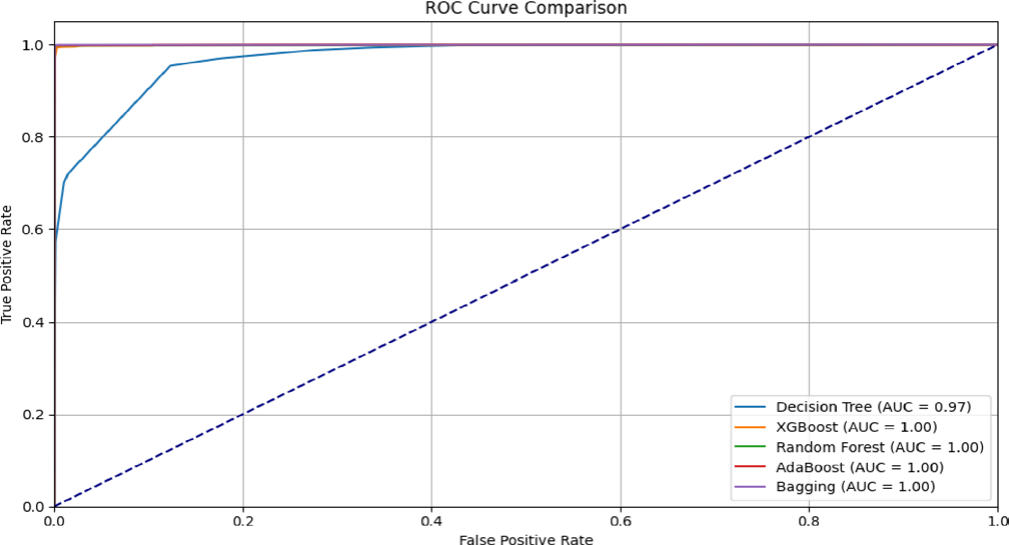
AUC-ROC performance of the proposed CG-GAO-with ensemble classifiers on the CICIDS17 dataset.

**Fig. 5:**
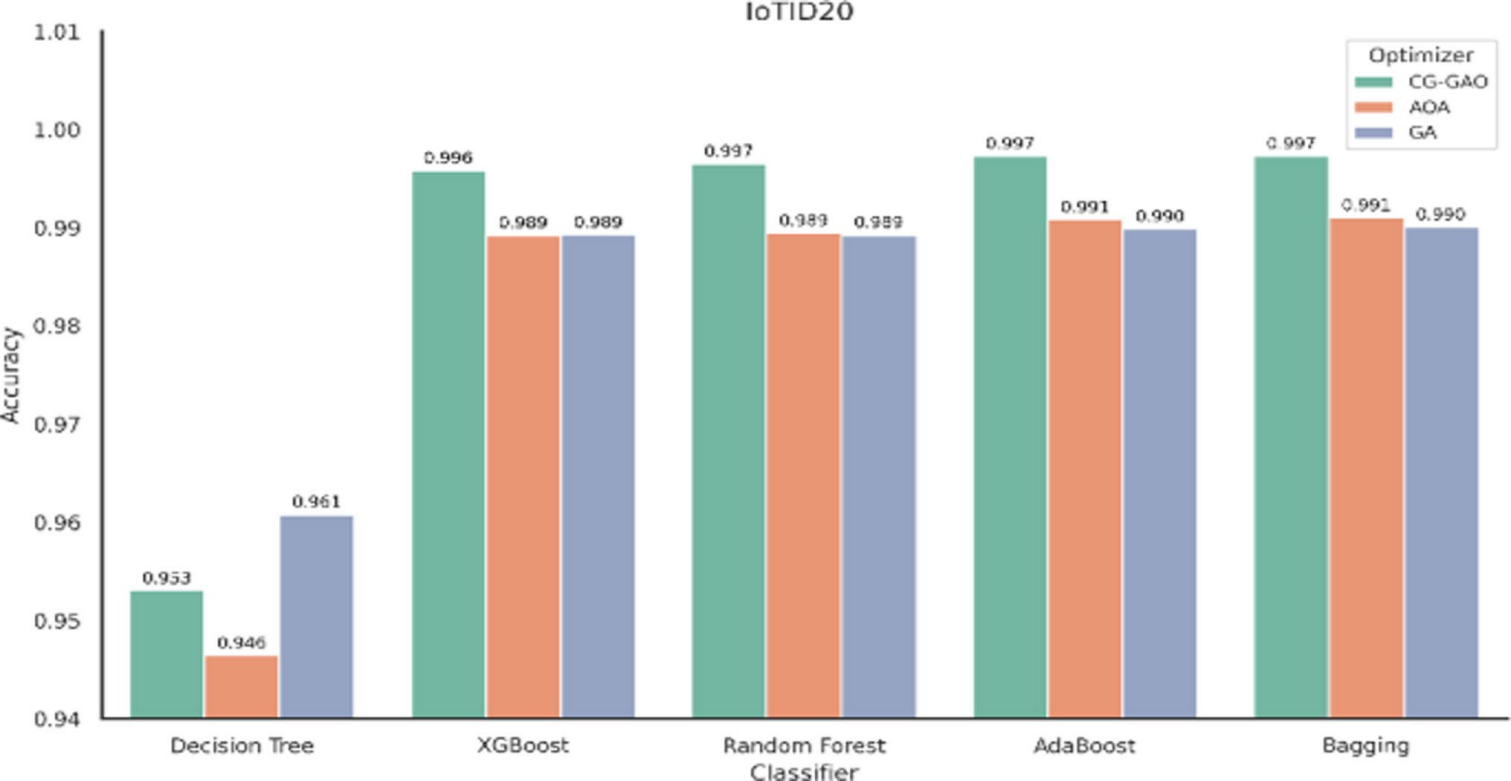
Accuracy of ensemble classifiers with feature selection techniques on IoTID20 dataset.

The learned samples from the HVS technique were input into the CG-GAO, AOA, and GA optimizers for feature selection. The setup for the CG-GAO algorithm is summarized in Table 4. Each optimizer was executed for 30 independent runs, owing to the stochastic nature of metaheuristics^46^, to ensure the reliability and statistical significance of the results, as depicted in Figs. 2, 3, 4, and 5.

**Table 4:** Configuration parameters of the proposed CG-GAO-based feature selection.

Framework parameter	Value
Population Initialization	Combined Cauchy & Gaussian Distributions (Binary Thresholding
Cauchy–Gaussian Mix Ratio	50% Cauchy, 50% Gaussian
Population Size	25
Max Iterations	100
Chromosome Encoding	Binary Vector (0/1 per feature)
Initial Mutation Rate	0.1 (increases to a max of 0.5 if no improvement is observed)
Mutation Strategy	Bit flip with minimum-feature constraint
Crossover Method	Single-Point Crossover
Selection Strategy	Roulette Wheel Selection based on fitness
Classifier	K-Nearest Neighbors (KNN), k = 5
Fitness Function	Fitness = λ × Classification Error + (1 − λ) × Feature Ratio, λ = 0.5
Exploration–Exploitation Balance	Controlled via AOA parameters (alpha = 0.5, mu = 0.5, e = 1)
AOA Parameters	MinMOA = 0.1, MaxMOA = 1, alpha = 0.5, mu = 0.5, e = 1
Hybrid Strategy	GA used during first 10% of iterations AOA used during remaining 90%
Binary Conversion	AOA updated individuals are thresholded: value > 0.5 → 1, else 0
Best Fitness Criteria	Minimum fitness value among population

**Fig. 6:**
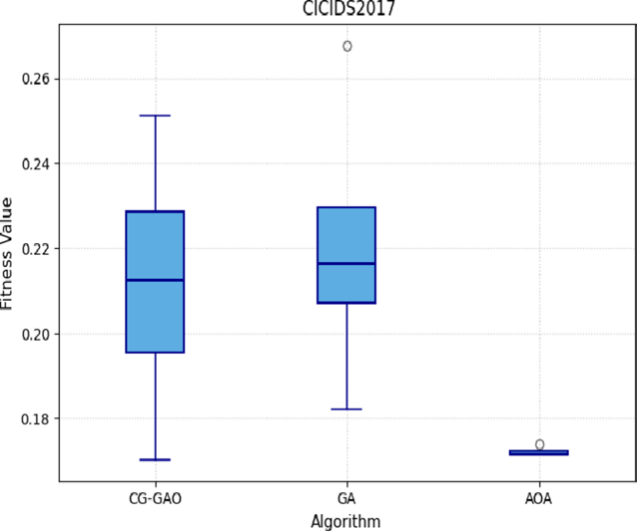
Box plot of optimizers on CICIDS2017-HVS sample.

**Fig. 7:**
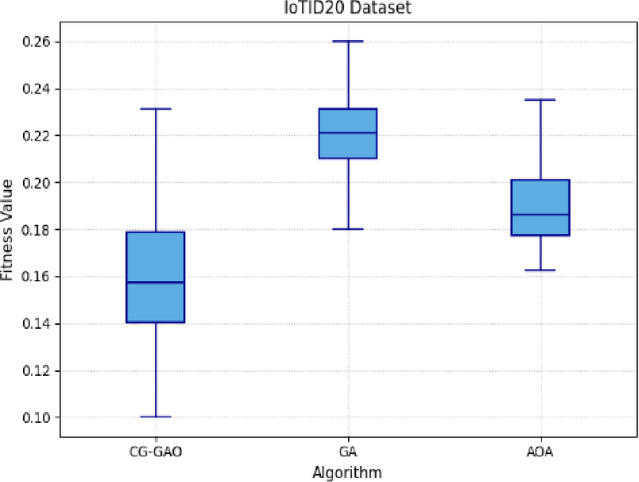
Box plot of optimizers on IoTID20-HVS sample.

Table 5 summarizes the best, worst, mean, and median fitness values, along with standard deviation, variance, and the number of selected features for each optimizer across both CICIDS2017 and IoTID20 datasets. In Supplementary file, Table S1 and Table S2 lists the features selected by CG-GAO, AOA, and GA from both datasets. The CG-GAO consistently achieved lower mean fitness values with relatively small variance, indicating more effective and stable feature selection. In contrast, GA showed higher mean values and greater variability, while AOA displayed consistent yet slightly sub-optimal fitness values. Figures 6 and 7 present the box plots of fitness values obtained across the independent runs, illustrating the distribution and stability of each optimizer’s performance.

**Table 5:** Metaheuristic algorithm performance on CICIDS2017 and IoTID20.

Dataset	Algorithm	Best	Worst	Mean	Median	Std	Var	Feature count
CICIDS2017	CG-GAO	**0.1701**	**0.2513**	**0.2039**	**0.2211**	**0.0267**	**0.0007**	**26**
	GA	0.1823	0.2678	0.2169	0.2156	0.0179	0.0003	30
	AOA	0.1715	0.174	0.1717	0.1715	0.0003	1.1e–07	28
IoTID 20	CG-GAO	**0.1000**	**0.2310**	**0.1537**	**0.1610**	**0.0307**	**0.0009**	**25**
	GA	0.1800	0.2600	0.2217	0.2200	0.0232	0.0005	29
	AOA	0.1625	0.2350	0.1896	0.1825	0.0265	0.0007	28

Significant values are in bold.

**Fig. 8:**
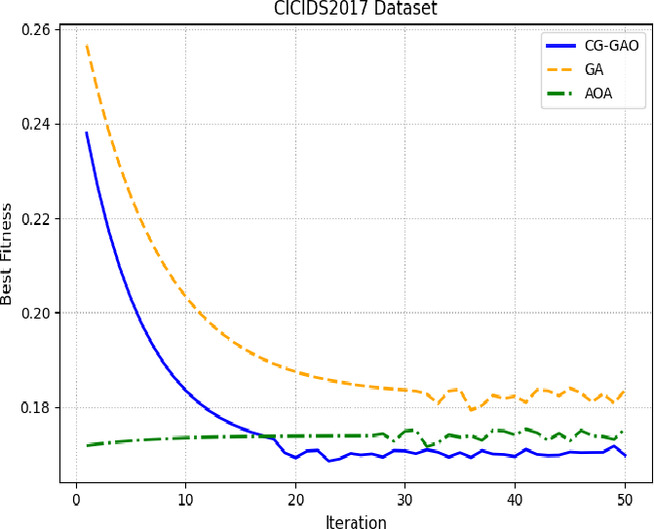
Fitness convergence of optimizers on CICIDS2017-HVS sample

**Fig. 9:**
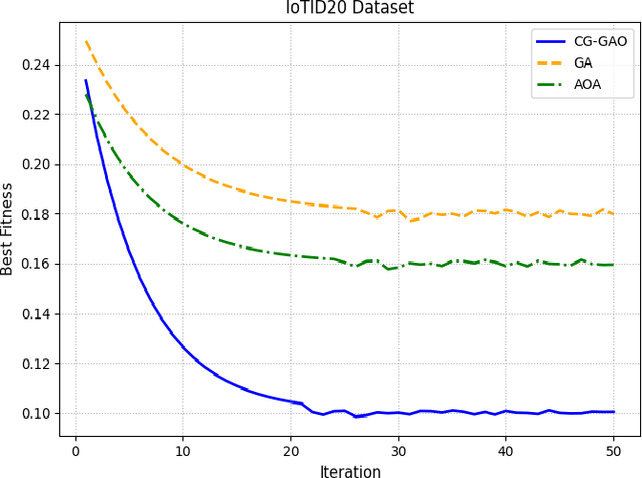
Fitness convergence of optimizers on IoTID20-HVS sample.

Figures 8 and 9 show the convergence behavior of CG-GAO, GA, and AOA across iterations for the CICIDS2017 and IoTID20 HVS samples. The CG-GAO optimizer reaches an optimal fitness value quickly and maintains stability throughout the iterations. The sensitivity analyses of CG-Gao for different hyperparameters were added in Supplementary, Table S3-S4. In contrast, GA exhibits oscillating behavior with poor convergence, while AOA converges early but to a sub-optimal level.

### Classification using ensemble classifiers

As outlined in Fig. 1, the features selected through hierarchical variance sampling (HVS) and optimized via CG-GAO, AOA, and GA algorithms were subsequently used to train a set of ensemble classifiers, including Decision Tree, XGBoost, Random Forest, AdaBoost, and Bagging. The hyperparameters of the Ensemble classifier are listed in Table 6.

**Table 6:** Hyper parameters for the ensemble classifiers.

Algorithm	Hyperparameters
Decision Tree	criterion = ‘gini’, max_depth = 5, random_state = 2, splitter = ‘random’
XGBoost	base_score = 0.3, n_estimators = 10
Random Forest	n_estimators = 1, random_state = 42
AdaBoost	base_estimator = DecisionTreeClassifier(), random_state = 42
Bagging	base_estimator = DecisionTreeClassifier(), random_state = 42

**Fig. 10:**
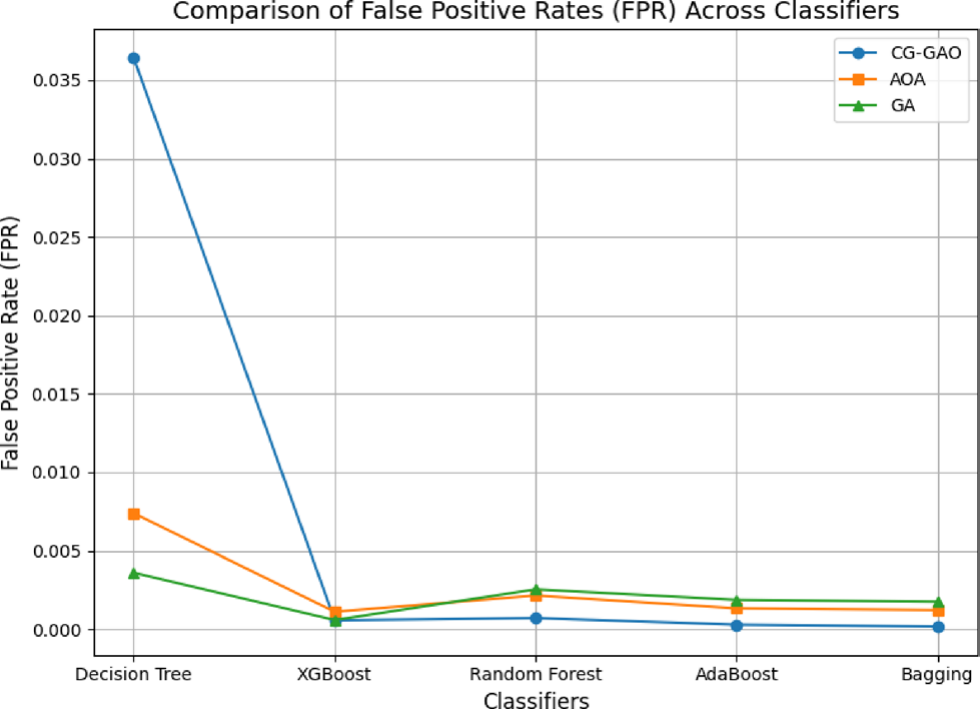
False positive rate of ensemble classifiers with feature selection techniques on IoTID20 dataset.

**Fig. 11:**
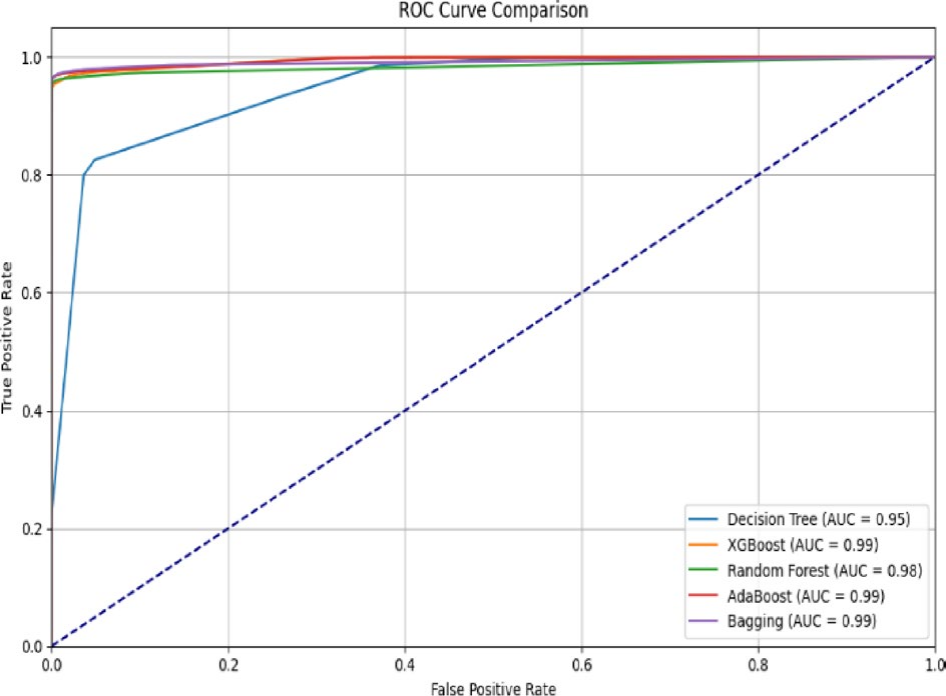
AUC-ROC performance of the proposed CG-GAO with ensemble classifiers on the IoTID20 dataset.

For the CICIDS2017 dataset, the IDS results are provided in Table 7, and the result graphs are illustrated in Figs. 2, 3 and 4. Among all combinations, CG-GAO with Bagging achieved the highest performance of precision 0.9980, recall 0.9982, F1-score 0.9981, and accuracy 0.9988. The proposed IDS with Bagging also showed an ROC AUC score of 0.9997, as in Fig. 3. In the IoTID20 dataset, performance metrics are detailed in Table 8 and result comparison graphs are added as Figs. 5, 10 and 11. Bagging achieved the highest accuracy, as shown in Fig. 5, with a precision of 0.9974, F1-score of 0.9884, and a low false positive rate of 0.000165, as in Fig. 10. In terms of AUC, Bagging achieved an ROC AUC of 0.9917, shown in Fig. 11. AdaBoost also performed well, with 0.9973 accuracy, 0.9965 precision, 0.9808 recall, 0.9885 F1-score, and a slightly higher FPR of 0.000285.

**Table 7:** Performance comparison of classifiers using CG-GAO, AOA, and GA optimizers on the CICIDS2017.

Classifier	Accuracy	Precision	Recall	F1_score	FPR
CG-GAO Decision Tree	0.932827	0.928703	0.852566	0.884044	0.014824
CG-GAO XGBoost	0.995306	0.994026	0.991132	0.992571	0.001971
CG-GAO Random Forest	0.998615	0.997565	0.998066	0.997815	0.001027
CG-GAO AdaBoost	0.998736	0.997836	0.998175	0.998005	0.000899
CG-GAO Bagging	**0.998809**	**0.998030**	**0.998212**	**0.998121**	**0.000801**
AOA Decision Tree	0.885676	0.931453	0.712576	0.764528	0.001419
AOA XGBoost	0.994988	0.995175	0.988980	0.992041	0.001093
AOA Random Forest	0.997954	0.996445	0.997103	0.996773	0.001491
AOA AdaBoost	0.998154	0.996846	0.997332	0.997089	0.001309
AOA Bagging	0.998273	0.997138	0.997415	0.997276	0.001166
GA Decision Tree	0.889018	0.875898	0.751398	0.792426	0.021220
GA XGBoost	0.994937	0.992179	0.991835	0.992007	0.003041
GA Random Forest	0.997122	0.995474	0.995443	0.995459	0.001783
GA AdaBoost	0.997620	0.996013	0.996479	0.996246	0.001636
GABagging	0.997763	0.996389	0.996551	0.996470	0.001447

Significant values are in bold.

**Table 8:** Performance comparison of classifiers using CG-GAO, AOA, and GA optimizers on the IoTID20.

Classifier	Accuracy	Precision	Recall	F1_score	FPR
CG-GAO-Decision Tree	0.953061	0.792755	0.881366	0.829873	0.036435
CG-GAO-XGBoost	0.995813	0.993712	0.97101	0.982090	0.000563
CG-GAO-Random Forest	0.996500	0.993136	0.977430	0.985136	0.000706
CG-GAO-AdaBoost	**0.997294**	0.996542	**0.980766**	**0.988507**	0.000285
CG-GAO-Bagging	**0.997278**	**0.997376**	**0.979826**	**0.988419**	**0.000165**
AoA-Decision Tree	0.946451	0.833135	0.631324	0.681931	0.007380
AoA-XGBoost	0.989208	0.985427	0.923122	0.951815	0.001110
AoA-Random Forest	0.989443	0.977958	0.932025	0.953653	0.002145
AoA-AdaBoost	0.990812	0.984934	0.937145	0.959611	0.001326
AoA-Bagging	0.991014	0.985872	0.937991	0.960501	0.001218
GA-Decision Tree	0.960774	0.927884	0.717585	0.783890	0.003596
GA-XGBoost	0.989277	0.989612	0.920052	0.951774	0.000580
GA-Random Forest	0.989251	0.975039	0.933204	0.953008	0.002538
GA-AdaBoost	0.989890	0.980313	0.933623	0.955592	0.001866
GA-Bagging	0.990060	0.981307	0.934141	0.956322	0.001747

Significant values are in bold.

In Table 9, the proposed Hybrid CG-GAO model performance is compared with recent metaheuristic-based feature selection approaches on the same CICIDS2017 and IoTID20 datasets. The Proposed CG-GAO shows better performance than the recent state of art methods.

**Table 9:** Comparison of proposed CG-GAO IDS with recent metaheuristic-based feature-selection IDS methods.

Year Ref	Metaheuristic feature selection	Dataset	Classifier	Accuracy	Feature Count	Detection time (s)
2024 ^13^	Hybrid OSMOGA(opposition-based learning + Slime Mold Algorithm + Genetic Algorithm)	CICIDS 2017	Artificial Neural Network	99.43%	19	17.26
2023 ^47^	Binary White Shark Optimizer with transfer function,cross over operators	CICIDS 2017	Modified K-Means clustering	99.14%	15	–
2024 ^49^	Binary Opposition Cellular Prairie dog optimization algorithm (BOC-PDO)	CICIDS 2017	KNN	99.21%	10	5.12
2022 ^14^	IDSBPSO + RF	IoTID20	Random Forest	99.84%	30	288
2025 ^48^	Binary Butterfly optimization Algorithm	IoTID20	LSTM	98.87	40	-
Proposed	Hybrid CG-GAO(Cauchy Gaussaian initialized Genetic Algortihm + Arithmetic Optimization Algorithm)	CICIDS2017 IoTID20	Bagging	**99.88%** and **99.72%**	**26** and **25**	**48** and **36**

Significant values are in bold.

### K-fold cross-validation

To assess the generalizability of the proposed CG-GAO intrusion detection framework, a fivefold cross-validation was performed on both the CICIDS2017 and IoTID20 datasets. The average performance across all folds is presented in Table 10. The results shows that the CG-GAO–Bagging model achieves the highest accuracy, recall, and F1-score on both datasets—99.88% accuracy, 99.82% recall, and 99.81% F1-score for CICIDS2017, and 99.73% accuracy, 97.98% recall, and 98.84% F1-score for IoTID20. Other models such as Random Forest, AdaBoost, and XGBoost also exhibit stable performance, confirming the efficacy of the proposed hybrid optimization approach.

**Table 10:** 5-Fold cross-validation results of CG-GAO models.

Dataset	Classifier	Accuracy	Precision	Recall	F1-score
CICIDS2017	DecisionTree	93.28	92.87	85.26	88.40
	XGBoost	99.53	99.40	99.11	99.26
	Random Forest	99.86	99.75	99.81	99.78
	AdaBoost	99.87	99.78	99.82	99.80
	Bagging	**99.88**	**99.80**	**99.82**	**99.81**
IoTID20	Decision Tree	95.31	79.28	88.14	82.98
	XGBoost	99.58	99.37	97.11	98.21
	Random Forest	99.65	99.31	97.74	98.51
	AdaBoost	99.72	99.65	98.07	98.85
	Bagging	**99.73**	**99.74**	**97.98**	**98.84**

Significant values are in bold.

### Ablation study of the proposed CG-GAO

An ablation study was performed to systematically assess the contribution of each component in the proposed CG-GAO pipeline using the Bagging classifier. The full framework integrates KD-tree based Hierarchical Variance Sampling (HVS), Cauchy–Gaussian population initialization, hybrid GA–AOA optimization, and the Bagging ensemble for classification.

Controlled variants were constructed by removing or modifying one component at a time, while keeping other settings identical. Each configuration was executed ten times, and the results (mean ± standard deviation) are presented in Table 11. The results indicate that the complete CG-GAO pipeline consistently achieves the best accuracy, recall, and F1-score. Replacing the hybrid initialisation with standard random initialisation leads to a larger decline in performance. The KD-tree HVS module contributes to efficiency by improving data compactness without degrading accuracy. The performance of IDS without feature selection shows the lowest performance.

**Table 11:** Ablation study results of the proposed CG-GAO IDS pipeline.

Components active	Dataset	Accuracy	Recall	F1-score	Training time (s)	Memory (MB)
**KD-tree + Cauchy–Gaussian Init + GA + AOA + Bagging (Full)**	CICIDS2017	**99.88 ± 0.06**	**99.82 ± 0.08**	**99.81 ± 0.07**	145 ± 9	370 ± 18
	IoTID20	**99.65 ± 0.08**	**97.89 ± 0.40**	**98.60 ± 0.30**	118 ± 7	245 ± 15
Cauchy–Gaussian Init + GA + AOA + Bagging (no KD-tree)	CICIDS2017	99.70 ± 0.08	99.68 ± 0.09	99.65 ± 0.08	185 ± 12	480 ± 22
	IoTID20	99.55 ± 0.10	97.70 ± 0.45	98.55 ± 0.35	160 ± 10	430 ± 20
KD-tree + Gaussian Init + GA + AOA + Bagging (no Cauchy)	CICIDS2017	99.67 ± 0.07	99.69 ± 0.08	99.68 ± 0.07	140 ± 9	410 ± 19
	IoTID20	99.65 ± 0.09	97.85 ± 0.42	98.60 ± 0.32	118 ± 7	380 ± 16
KD-tree + Cauchy Init + GA + AOA + Bagging (no Gaussian)	CICIDS2017	99.74 ± 0.07	99.68 ± 0.08	99.67 ± 0.07	142 ± 9	412 ± 18
	IoTID20	99.62 ± 0.09	97.80 ± 0.43	8.55 ± 0.33	119 ± 8	378 ± 16
KD-tree + Standard Init + GA + AOA + Baggig	CICIDS2017	98.65 ± 0.12	98.60 ± 0.14	98.58 ± 0.13	138 ± 9	408 ± 20
	IoTID20	99.50 ± 0.13	97.55 ± 0.50	98.47 ± 0.40	116 ± 8	372 ± 18
KD-tree + Standard Init + GA + Bagging (no AOA)	CICIDS2017	98.87 ± 0.11	98.54 ± 0.13	98.55 ± 0.12	130 ± 8	425 ± 21
	IoTID20	97.12 ± 0.25	92.65 ± 0.90	94.87 ± 0.70	110 ± 7	368 ± 17
KD-tree + Standard Init + AOA + Bagging (no GA)	CICIDS2017	97.71 ± 0.14	97.62 ± 0.15	97.60 ± 0.15	125 ± 8	495 ± 24
	IoTID20	97.51 ± 0.20	92.10 ± 0.85	94.46 ± 0.65	108 ± 7	365 ± 16
Full Dataset + Bagging (no FS or optimization)	CICIDS2017	98.25 ± 0.10	98.20 ± 0.12	98.15 ± 0.11	120 ± 8	490 ± 22
	IoTID20	**99.20** ± 0.13	97.00 ± 0.17	98.02 ± 0.40	105 ± 6	363 ± 14

Significant values are in bold.

### Statistical evaluation of optimisers

To validate the effectiveness of the proposed CG-GAO algorithm compared to the standard Genetic Algorithm (GA) and Arithmetic Optimization Algorithm (AOA), we employed a non-parametric statistical testing approach. The Friedman test was applied with degrees of freedom (*df* = k − 1 = 2), where *k* is the number of algorithms. The test ranks each algorithm’s performance across datasets, and the Friedman statistic (χ^2^) quantifies whether the mean ranks differ significantly. The Nemenyi test was then used for pairwise comparisons, computing q-statistics, p-values, and the critical difference (CD) — the minimum difference in average ranks is required to prove significance. To overcome the multiple comparison problem, the Nemenyi test adjusts significance thresholds based on the number of pairwise tests. All tests were performed at a 95% confidence level (α = 0.05).

**Fig. 12:**
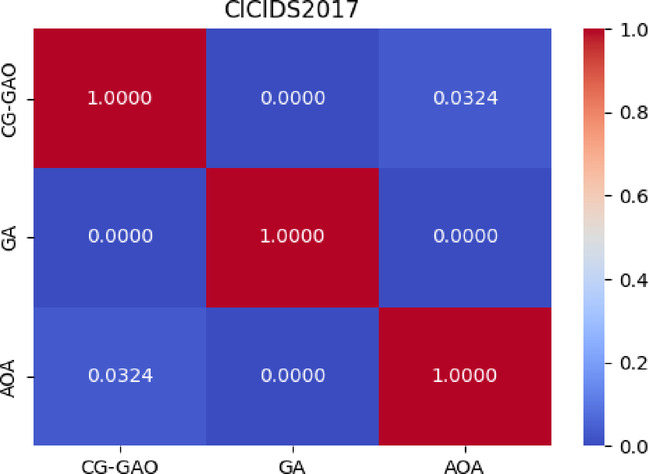
Pairwise *p* values from Nemenyi post-hoc test for CICIDS2017.

**Fig. 13:**
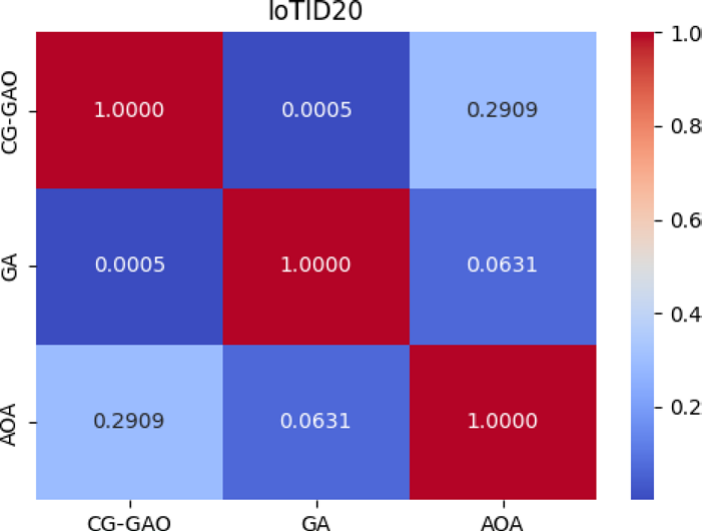
Pairwise *p* values from Nemenyi post-hoc test for IoTID20.

As in Table 12, For the CICIDS2017 dataset, the Friedman test yielded χ^2^(2) = 61.35, *p* < 0.0001, with average ranks CG-GAO = 1.12, AOA = 2.23, and GA = 2.65. The critical difference (CD) was 0.825, and the Nemenyi test confirmed that CG-GAO significantly outperformed GA (q = 5.67, *p* < 0.001) and AOA (q = 2.81, *p* = 0.032).For the IoTID20 dataset, the Friedman test reported χ^2^(2) = 14.25, *p* = 0.0008, with average ranks CG-GAO = 1.35, AOA = 2.23, and GA = 2.42, and CD = 0.905. The Nemenyi test indicated that CG-GAO significantly outperformed GA (q = 4.25, *p* = 0.0005), while the difference between CG-GAO and AOA (q = 1.05, *p* = 0.291) was not statistically significant, suggesting comparable performance in that scenario. Figures 12 and 13 present heatmaps of the Nemenyi post-hoc test *p* values for the CICIDS2017 and IoTID20 datasets, respectively. These visualisations highlight the statistical significance of performance differences between algorithm pairs, supporting the numerical results in Table 12.

**Table 12:** Friedman and Nemenyi test results on CICIDS2017 and IoTID20.

Dataset	Friedman ${\chi }^{2}$	*p* value	Avg.Rank (CG-GAO)	Avg.Rank (AOA)	Avg.Rank (GA)	CD	CG-GAO versus GA	CG-GAO versus AOA	GA versus AOA
CICIDS2017	61.35	< 0.001	1.12	2.23	2.65	0.85	5.67, < 0.0001	2.81, 0.0324	3.45, < 0.0001
IoTID20	14.25	0.0008	1.35	2.23	2.42	0.95	4.25, 0.0005	1.05, 0.2909	1.63,0.0631

### Complexity analysis of hierarchical variance sampling with hybrid GA-AOA feature selection

The proposed method combines Hierarchical Variance Sampling (HVS) with a Hybrid CG-GAO algorithm for efficient feature selection in high-dimensional data.

HVS Phase: Calculates variance in O(n⋅b) where n is Number of data instances and b is Number of features whose variance is calculated for splitting and recursively partitions data using a KD-Tree until subset size t(bucket size), Since the KD-Tree has a depth of log(n/t) the overall complexity becomes:

$${\text{O}}(({\text{n}} \cdot {\text{b}}){\text{log}}\left( {{\text{n}}/{\text{t}}} \right))$$

Since HVS samples based on feature variance, it selects fewer yet more informative instances compared to random or stratified sampling. This reduces input size for feature selection while maintaining performance.

Feature Selection Phase: Hybrid CG-GAO uses Cauchy–Gaussian initialization, GA for exploration, and AOA for exploitation. The time complexity is driven by the number of individuals N, the number of iterations ${I}_{t}$, and the cost per evaluation, approximated as:

$$O(N.{I}_{t}.(0.1a+0.9b))$$

where a is the Complexity of genetic operations and b is the Complexity of AOA arithmetic updates. Compared to conventional feature selection methods, which often rely on exhaustive search (high complexity, typically $O({2}^{b})$ for b features) or greedy algorithms (complexity of $O(n.{b}^{2})$ in some cases), the HVS combined with CG-GAO is relatively low in complexity, particularly for large datasets Table 13 shows a comparison of the CG-GAO, GA, and AOA optimization algorithms based on their computational complexity, iteration strategy, number of function evaluations (FFEs), and overall resource usage. The CG-GAO hybrid method provides a good balance between speed and cost by combining both GA and AOA steps. In contrast, GA has the highest cost because it runs a full genetic search, while AOA is the least costly but may stop early before finding the best solution. A detailed breakdown of the computational cost for each stage of the proposed framework—HVS sampling, CG-GAO feature selection, and ensemble classification—across both datasets is provided in Table S5 in supplementary file.

**Table 13:** Comparative computational complexity and resource cost of metaheuristic optimizers.

Algorithm	Optimization method	Complexity	FFEs	Relative cost	Advantages
CG-GAO	GA (10%) AOA (90%)	$O(N.{I}_{t}.(0.1a+0.9b))$ (a > b)	Moderate to high	Balanced	Best accuracy; efficient blend of global and local search
GA	GA only	$O(N.{I}_{t}.a)$	High	Highest	Pure exploration; lacks local refinement
AOA	AoA only	$O(N.{I}_{t}.b)$	Low	Lowest	Fastest; lower accuracy in complex feature spaces

## Conclusion

This research presents a novel sampling and hybrid Feature optimization techniques to enhance anomaly detection efficiency and reduce the computational burden. The proposed approach Cauchy–Gaussian Genetic-Arithmetic Optimizer (CG-GAO) based active feature selection integrated with ensemble machine learning classifiers supports effective binary classification. The Proposed IDS lowers computation cost and improves population diversity, convergence speed, and feature relevance, resulting in effective dimensionality reduction.

Experiments on CICIDS2017 and IoTID20 datasets showed that CG-GAO with Bagging achieved the best accuracy—99.89% and 99.72%, respectively, with high precision, recall, and F1-scores and minimal false positive rates. The statistical significance of the proposed framework is confirmed by Friedman test with *p* < 0.0001 for CICIDS2017, *p* = 0.0008 for IoTID20, followed by Nemenyi post-hoc analysis, where CG-GAO showed significant performance over GA (*p* < 0.001) and AoA (*p* = 0.032 on CICIDS2017. Despite its promising performance, the proposed CG-GAO framework has certain limitations. The framework has been evaluated only on CICIDS2017 and IoTID20 datasets, and its effectiveness on broader, more diverse IoT and real-world traffic remains to be validated. While the proposed method demonstrates strong performance, this study does not explicitly address certain practical deployment aspects such as real-time operation, generalisation under dynamic network conditions, and concept drift. These remain important considerations for fully operational IDS environments. In future work, we plan to extend the CG-GAO framework to additional IoT and real-world datasets to further assess its generalizability, evaluate its robustness against zero-day attacks, and incorporate Explainable AI (XAI) for improved interpretability. We also intend to develop and test mechanisms suitable for real-time IDS deployment, ensuring efficient adaptation to evolving threat patterns.

## Supplementary Information

Below is the link to the electronic supplementary material.

Supplementary Material 1

## Data Availability

The datasets used in this research are publicly available. The CICIDS 2017 dataset can be accessed at [https://www.unb.ca/cic/datasets/ids-2017.html](https:/www.unb.ca/cic/datasets/ids-2017.html) , and the IoTID20 dataset is available at [https://sites.google.com/view/iot-network-intrusion-dataset/home](https:/sites.google.com/view/iot-network-intrusion-dataset/home) .
