# Supplementary material for: Optimized intrusion detection for IoT networks using Cauchy–Gaussian hybrid evolutionary feature selection
Source: Sci Rep. 2025 Dec 20;16:501. doi: 10.1038/s41598-025-29884-5 (PMC12775014; doi:10.1038/s41598-025-29884-5)
Supplement: Supplementary file 1 — Supplementary Material 1 [file 41598_2025_29884_MOESM1_ESM.docx]

Optimized Intrusion Detection for IoT Networks Using Cauchy-Gaussian Hybrid Evolutionary Feature Selection-SUPPLEMENTARY

Saranya.T^1^[0009-0006-1202-9486] and Indra Priyadharshini. S^2*^[0000-0002-0891-1605]

^1^ Research Scholar, Computer Science and Engineering, Chennai,600127, India.

^2^ Assistant Professor, Computer Science and Engineering, Chennai,600127, India

indra.priyadharshini@vit.ac.in

# **The Principal Component Analysis (PCA) projections of sampled data**

The Principal Component Analysis (PCA) projections before and after HVS sampling, illustrated in Figure S1to S4, demonstrate that the sampling process effectively preserves the intrinsic data structure of the original datasets.

| 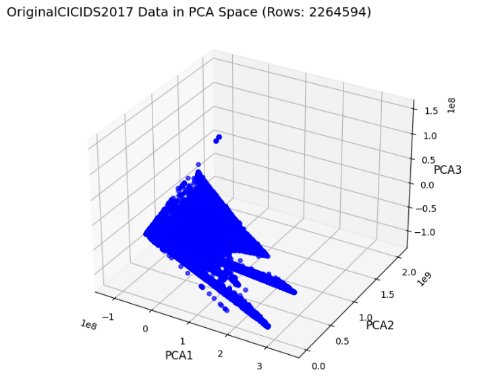  **Fig S1**. PCA Projection of Original Data Samples- CICIDS2017 | 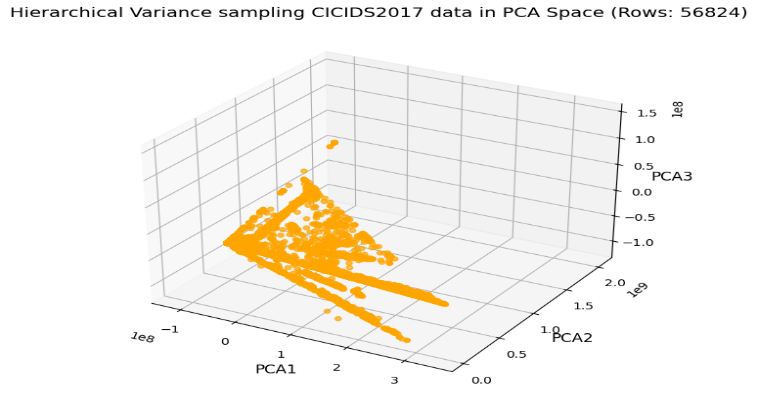  **Fig S2**. PCA Projection of HVS-Selected Samples – CICIDS2017 |
| --- | --- |
| 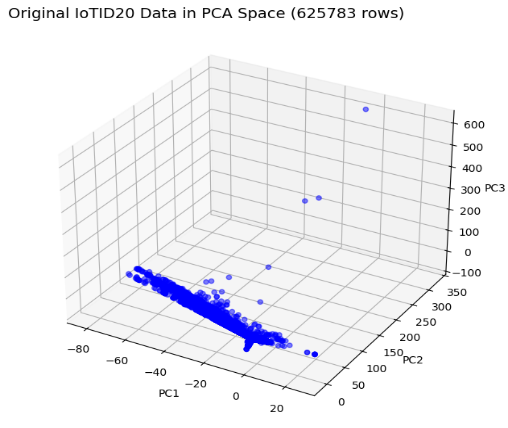  **Fig S3.** PCA Projection of Original Data Samples- IoTID20 | 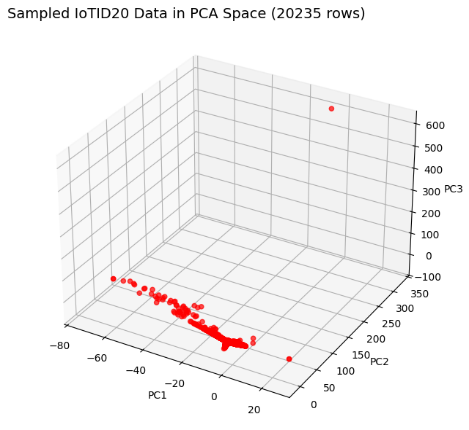  **Fig S4**.. PCA Projection of HVS-Selected Samples-IoTID20 |

**Features Selected by Feature Selection Optimizers**

Table S1 . Features chosen from the CICIDS2017 dataset using a feature selection algorithm and sampling

| **CG-GAO-HVS** | Total Backward Packets,Total Length of Bwd Packets, Fwd Packet Length Min, Fwd Packet Length Mean, Flow IAT Min, Bwd IAT Std, Bwd IAT Min, Bwd URG Flags, Fwd Header Length, Packet Length Mean, FIN Flag Count, PSH Flag Count, ACK Flag Count,URG Flag Count, ECE Flag Count, Down/Up Ratio, Fwd Avg Packets/Bulk, Subflow Fwd Packets, Init_Win_bytes_forward,Init_Win_bytes_backward, Active Mean, Active Std, Active Min, Idle Mean, Idle Max, Idle Min. |
| --- | --- |
| **AoA** **-HVS** | Flow Duration, Total Fwd Packets, Fwd Packet Length Max, Fwd Packet Length Mean, Bwd Packet Length Mean, Flow Bytes/s, Fwd IAT Mean, Bwd IAT Total, Bwd IAT Std, Fwd PSH Flags, Fwd URG Flags, Min Packet Length, Packet Length Variance, FIN Flag Count, RST Flag Count, ACK Flag Count, Average Packet Size, Avg Fwd Segment Size, Fwd Avg Packets/Bulk, Bwd Avg Bytes/Bulk, Bwd Avg Bulk Rate, Subflow Fwd Bytes, Subflow Bwd Bytes, Subflow Bwd Packets, Init_Win_bytes_forward, Init_Win_bytes_backward, Active Mean, Idle Max, Idle Min, Flow Packets/s |
| **GA-HVS** | Destination Port, Total Fwd Packets, Total Backward Packets, Fwd Packet Length Min, Fwd Packet Length Mean, Fwd Packet Length Std, Flow Bytes/s, Flow IAT Mean, Flow IAT Std, Flow IAT Max, Fwd IAT Max, Fwd IAT Min, Bwd IAT Max, Fwd URG Flags, Bwd URG Flags, Packet Length Mean, RST Flag Count, URG Flag Count, Down/Up Ratio, Bwd Avg Bytes/Bulk, Bwd Avg Bulk Rate, Subflow Fwd Bytes, Subflow Bwd Packets, Subflow Bwd Bytes, min_seg_size_forward, ACK Flag Count, Packet Length Std, Avg Bwd Segment Size |

Table S2 . Features chosen from IIoT20 dataset using Feature selection Algorithm and sampling:

| **CG-GAO-HVS** | Dst_Port, Protocol, TotLen_Bwd_Pkts, Fwd_Pkt_Len_Min, Fwd_Pkt_Len_Std,Bwd_Pkt_Len_Max, Bwd_Pkt_Len_Min, Bwd_Pkt_Len_Std, Flow_Byts/s,  Fwd_IAT_Std, Fwd_IAT_Min, Fwd_URG_Flags, Bwd_Header_Len, Bwd_Pkts/s,Pkt_Len_Max, SYN_Flag_Cnt, CWE_Flag_Count, Bwd_Seg_Size_Avg,Fwd_Byts/b_Avg, Subflow_Bwd_Pkts, Subflow_Bwd_Byts,Fwd_Act_Data_Pkts, Fwd_Seg_Size_Min, Active_Max, Idle_Mean |
| --- | --- |
| **AoA- HVS** | Src_Port, Fwd_Pkt_Len_Max, Fwd_Pkt_Len_Mean, Bwd_Pkt_Len_Std,Flow_Pkts/s, Flow_IAT_Max, Fwd_IAT_Tot, Fwd_IAT_Mean, Fwd_IAT_Std,Fwd_IAT_Max, Bwd_IAT_Mean, Fwd_URG_Flags, Bwd_URG_Flags, Pkt_Len_Var,FIN_Flag_Cnt, CWE_Flag_Count, Bwd_Seg_Size_Avg, Fwd_Byts/b_Avg,Bwd_Byts/b_Avg, Bwd_Blk_Rate_Avg, Subflow_Bwd_Pkts, Init_Fwd_Win_Byts,Fwd_Act_Data_Pkts, Fwd_Seg_Size_Min, Active_Mean, Idle_Max, Idle_Mean,Pkt_Len_Min, TotLen_Fwd_Pkts |
| **GA-HVS** | Flow_Duration, TotLen_Bwd_Pkts, Fwd_Pkt_Len_Max, Bwd_Pkt_Len_Min,Bwd_Pkt_Len_Mean, Flow_Byts/s, Flow_IAT_Mean, Fwd_IAT_Max, Bwd_IAT_Std,Bwd_IAT_Min, Bwd_URG_Flags, Bwd_Header_Len, Fwd_Pkts/s, Bwd_Pkts/s,Pkt_Len_Min, Pkt_Len_Max, Pkt_Len_Var, Fwd_Byts/b_Avg, Bwd_Byts/b_Avg,Subflow_Fwd_Pkts, Subflow_Fwd_Byts, Init_Bwd_Win_Byts,Fwd_Act_Data_Pkts, Idle_Max, Idle_Min, Init_Fwd_Win_Byts,Pkt_Len_Mean, Bwd_Pkt_Len_Std |

# **Sensitivity Analysis of Hyperparameters**

To examine the sensitivity of the proposed CG-GAO algorithm to the choice of hyperparameters key hyperparameters were varied while others were held constant. Each configuration was tested on the CICIDS2017 and IoTID20 dataset using the KNN classifier (k = 5), and results were averaged over 10 independent runs as in table C1. From the sensitivity analysis, the mutation rate and fitness balance parameter (λ) were found to be the sensitive hyperparameters, as small changes in their values affects the fitness score and number of selected features.

**Table S3: Sensitivity Analysis of Hyperparameters**

| **Parameter** | **Test**  **Range** | **Best Value** | **Dataset** | **Best Fitness Val** | **Worst Finess Value** | **Feature Count (Avg)** |
| --- | --- | --- | --- | --- | --- | --- |
| **Population Size** | 10–50 | 25 | CICIDS2017 | 0.1701 | 0.2513 | 26 |
|  |  |  | IoTID20 | 0.1000 | 0.2310 | 25 |
| **Mutation Rate** | 0.1–0.6 | 0.1–0.5 | CICIDS2017 | 0.1701 | 0.2450 | 26–29 |
|  |  |  | IoTID20 | 0.1000 | 0.2280 | 25–28 |
| **Crossover Probability** | 0.6–0.9 | 0.8 | CICIDS2017 | 0.1710 | 0.2280 | 27 |
|  |  |  | IoTID20 | 0.1020 | 0.2250 | 25–26 |
| **λ (Fitness Balance)** | 0.3–0.7 | 0.5 | CICIDS2017 | 0.1701 | 0.2200 | 24–29 |
|  |  |  | IoTID20 | 0.1000 | 0.2100 | 23–28 |
| **Cauchy–Gaussian Mix Ratio** | 25/75–75/25 | 50/50 | CICIDS2017 | 0.1701 | 0.2039 | 26 |
|  |  |  | IoTID20 | 0.1000 | 0.1537 | 25 |

**Sensitivity of Classifier Choice in Wrapper Fitness**

To evaluate the sensitivity of CG-GAO to the choice of classifier within the wrapper fitness function, KNN (k = 5) was replaced with alternative classifiers—Random Forest (RF), Decision Tree (DT), and Logistic Regression (LR). All other CG-GAO parameters were kept constant, and the Bagging ensemble was used for the final evaluation to ensure a fair comparison. The results in Table S4 show that KNN-based feature selection yields the highest accuracy and F1-score on both datasets.

Table S4: Classifier Sensitivity in Wrapper Fitness Function

| **Wrapper Classifier** | **Dataset** | **Accuracy (%)** | **Recall (%)** | **F1-Score (%)** | **Training Time (s)** |
| --- | --- | --- | --- | --- | --- |
| **KNN** | CICIDS2017 | 99.88 ± 0.06 | 99.82 ± 0.08 | 99.81 ± 0.07 | 145 ± 9 |
|  | IoTID20 | 99.65 ± 0.08 | 97.89 ± 0.40 | 98.60 ± 0.30 | 118 ± 7 |
| Random Forest | CICIDS2017 | 99.74 ± 0.07 | 99.68 ± 0.09 | 99.66 ± 0.08 | 162 ± 10 |
|  | IoTID20 | 99.60 ± 0.09 | 97.82 ± 0.42 | 98.55 ± 0.32 | 130 ± 8 |
| Decision Tree | CICIDS2017 | 99.40 ± 0.10 | 99.25 ± 0.11 | 99.23 ± 0.11 | 138 ± 9 |
|  | IoTID20 | 99.15 ± 0.12 | 97.10 ± 0.46 | 98.05 ± 0.38 | 120 ± 7 |
| Logistic  Regression | CICIDS2017 | 98.95 ± 0.15 | 98.70 ± 0.16 | 98.68 ± 0.15 | 128 ± 8 |
|  | IoTID20 | 98.75 ± 0.18 | 97.00 ± 0.50 | 97.85 ± 0.42 | 115 ± 7 |

Empirical Runtime Analysis

**Table S5**.Average runtime for each stage of the pipeline

| **Dataset** | **Stage** | **Runtime** |
| --- | --- | --- |
| CICIDS2017 | HVS Sampling | ~0.20 h |
|  | CG-GAO Feature Selection | ~10.5 h |
|  | Ensemble Classification | ~.45 h |
| IoTID20 | HVS Sampling | ~0.15 h |
|  | CG-GAO Feature Selection | ~6.5 h |
|  | Ensemble Classification | ~0.25 h |

The reported runtimes in Table S1 represent the total computation time (training and testing) for each stage, averaged over multiple runs. The runtime analysis shows that the CG-GAO feature selection stage dominates computation, while HVS efficiently reduces the dataset size, enabling the ensemble classifiers to operate on a smaller, representative feature set. Despite higher computational cost, this pipeline achieves dimensionality reduction and robust IDS performance, making it suitable for practical deployment after offline training.
